# Oxygen Isotope Fractionation of O_2_ Consumption through Abiotic Photochemical Singlet Oxygen Formation Pathways

**DOI:** 10.1021/acsenvironau.4c00107

**Published:** 2025-01-22

**Authors:** Sarah G. Pati, Lara M. Brunner, Martin Ley, Thomas B. Hofstetter

**Affiliations:** † Department of Environmental Geosciences, Centre for Microbiology and Environmental Systems Science, University of Vienna, Vienna 1090, Austria; ‡ Department of Environmental Sciences, University of Basel, Basel 4056, Switzerland; § Eawag, Swiss Federal Institute of Aquatic Science and Technology, Dübendorf 8600, Switzerland; ∥ Institute of Biogeochemistry and Pollutant Dynamics (IBP), ETH Zürich, Zürich 8092, Switzerland

**Keywords:** oxygen isotope ratio, biogeochemical O_2_ cycling, photochemical oxygen activation, singlet oxygen, isotope fractionation

## Abstract

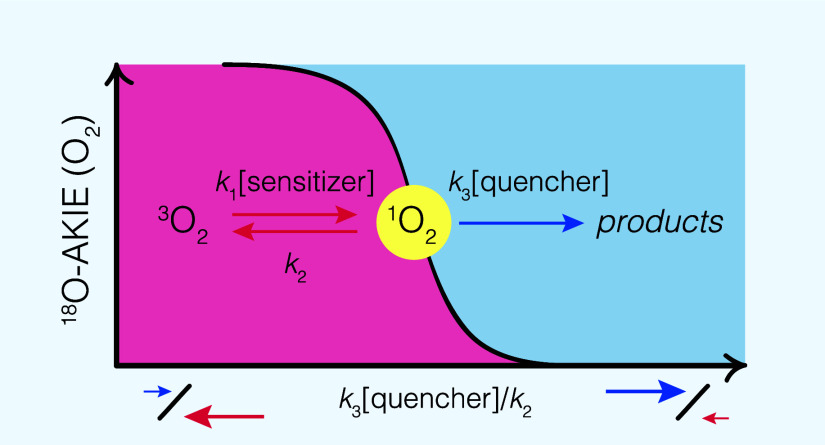

Oxygen isotope ratios of O_2_ are important tracers for assessing biological activity in biogeochemical processes in aquatic environments. In fact, changes in the ^18^O/^16^O and ^17^O/^16^O ratios of O_2_ have been successfully implemented as measures for quantifying photosynthetic O_2_ production and biological O_2_ respiration. Despite evidence for light-dependent O_2_ consumption in sunlit surface waters, however, photochemical O_2_ loss processes have so far been neglected in the stable isotope-based evaluation of oxygen cycling. Here, we established the magnitude of the O isotope fractionation for abiotic photochemical O_2_ elimination through formation of singlet O_2_, ^1^O_2_, and the ensuing oxygenation and oxidation reactions with organic compounds through experiments with rose bengal as the ^1^O_2_ sensitizer and three different amino acids and furfuryl alcohol as chemical quenchers. Based on the kinetic analysis of light-dependent O_2_ removal in the presence of different quenchers, we rationalize the observable O isotope fractionation of O_2_ and the corresponding, apparent ^18^O kinetic isotope effects (^18^O-AKIE) with a pre-equilibrium model for the reversible formation of ^1^O_2_ and its irreversible oxygenation reactions with organic compounds. While ^18^O-AKIEs of oxygenation reactions amount to 1.03, the O isotope fractionation of O_2_ decreased to unity with increasing ratio of the rates of oxygenation reaction of ^1^O_2_ vs ^1^O_2_ decay to ground state oxygen, ^3^O_2_. Our findings imply that O isotope fractionation through photochemical O_2_ consumption with isotope enrichment factors, ^18^O-ϵ, of up to −30‰ can match contributions from biological respiration at typical dissolved organic matter concentrations of lakes, rivers, and oceans and should, therefore, be included in future evaluations of biogeochemical O_2_ cycling.

## Introduction

Dissolved oxygen, O_2_, is a crucial chemical species in biogeochemical cycles of aquatic environments, and its concentration is a critical parameter used for assessing ecosystem health. Understanding the main processes affecting the dynamics of dissolved O_2_ concentrations in lakes, rivers, and oceans, namely photosynthesis, respiration, and gas–water exchange, is therefore key to evaluate functions of pristine and human-impacted ecosystems.^1^ The quantification of contributions of these three processes of O_2_ production, consumption, and exchange to the overall O_2_ cycling is difficult to achieve from the monitoring and modeling of O_2_ concentration dynamics. To that end, information from the ratios of the three stable O isotopes, ^16^O, ^17^O, and ^18^O, as ^18^O/^16^O and ^17^O/^16^O in so-called triple oxygen isotope analysis are increasingly exploited to disentangle contributions of the three main processes of biogeochemical O_2_ cycling.^1,2^

**Figure 1:**
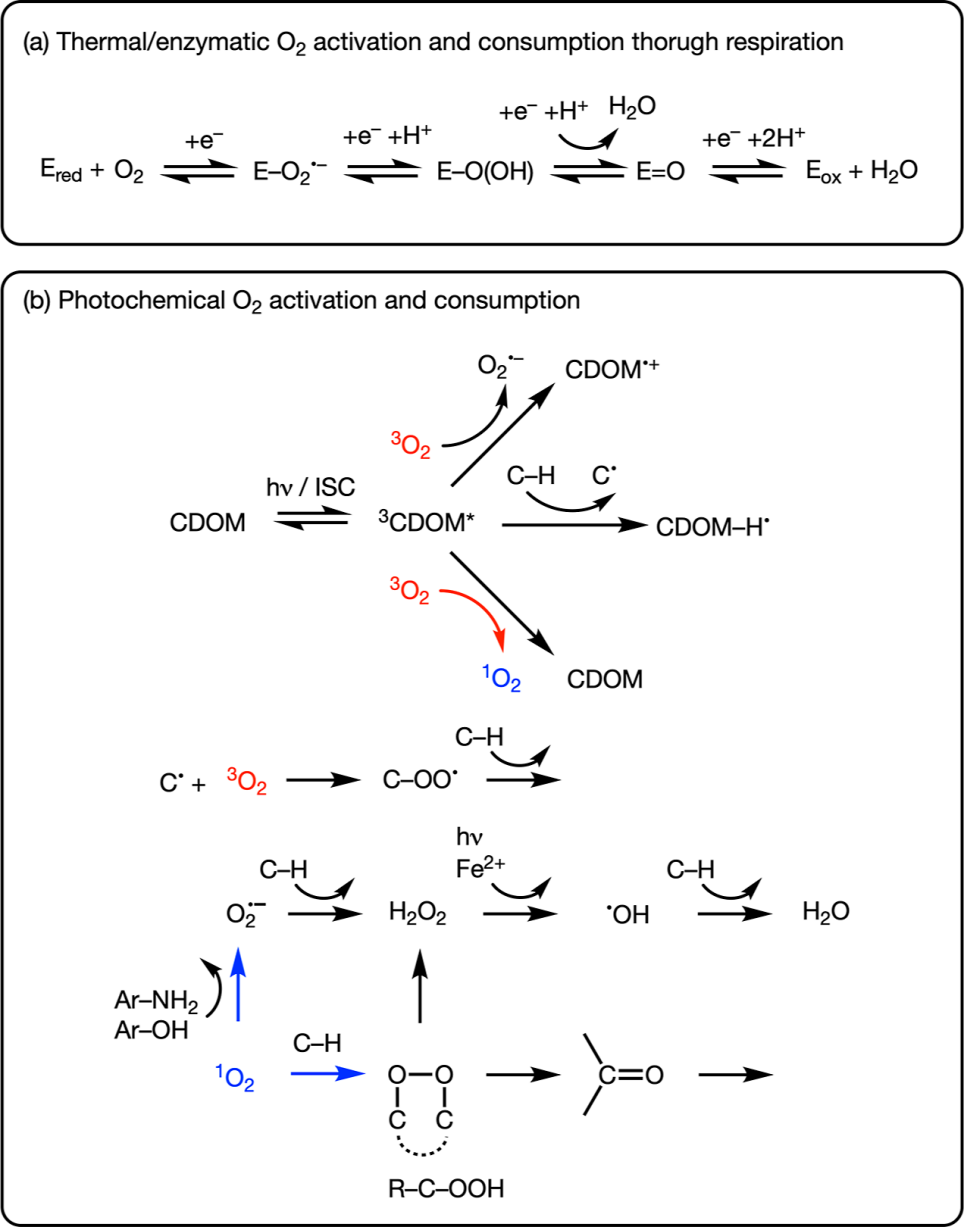
Schematic survey of processes and selected reaction steps responsible for O_2_ consumption in aquatic environments. (a) Respiration is the enzyme-catalyzed activation of O_2_ (where *E* stands for enzyme) and stepwise 4-electron reduction to H_2_O, where *E*_red_ and *E*_ox_ stand for oxidoreductases in their reduced and oxidized form, respectively. Reduction equivalents for O_2_ reduction from oxidation of organic substrates and cofactors are represented as electrons (e^–^). (b) Abiotic photochemical O_2_ consumption is shown here exemplary for processes induced by light-absorption and intersystem crossing of chromophoric dissolved organic matter (CDOM) to its excited triplet states (^3^CDOM*). C–H and C^•^ stand for organic compounds and carbon-centered radicals, respectively. Reactions of ^3^CDOM* with ^3^O_2_ act as source of ^1^O_2_ (red arrow). ^1^O_2_ loss processes contributing to photochemical O_2_ consumption (blue arrows) include oxygenation of organic compounds and one-electron reduction, for example, with phenols (Ar–OH) and organic amines (Ar–NH_2_), to O_2_^•–^.^3−6^

In fact, oxygen isotope-based analyses have been successfully implemented in combination with dissolved O_2_ dynamics and/or O_2_–Ar ratios to quantify gross photosynthetic O_2_ production, net community O_2_ production, and photosynthesis rates in both marine and freshwater systems.^1,7−14^ These isotope-based assessment of O_2_ formation and consumption are based on the simplified assumption that respiration, that is the formal 4-electron reduction of O_2_ to H_2_O (Figure 1a), is the dominating O_2_ sink term. Any other O_2_ loss processes have thus far been neglected in the evaluation of O isotope ratios. However, this view is being challenged through evidence showing that in sunlit surface waters, light-dependent O_2_ consumption can occur at similar or higher rates than the light-independent (i.e., “dark”) respiration of O_2_.^15−21^ Such O_2_ consumption by photochemical reactions in marine waters is estimated to be of the same magnitude as photosynthetic O_2_ production by phytoplankton.^19^ Light-dependent O_2_ consumption can occur through biological and abiotic processes. Biological, light-dependent O_2_ loss takes place through reduction of O_2_ initiated by excess electrons from photosystem II and, similar to respiration, leads to the formation of water.^20,22^ Abiotic, photochemical processes, by contrast, proceed through several, chemically distinct paths of O_2_ elimination (Figure 1b).

Abiotic photochemical O_2_ consumption is initiated by the light-absorption of chromophoric dissolved organic matter (CDOM), nitrate, nitrite, or transition-metals and ensuing reactions with O_2_. These reactions typically lead to the production of reactive oxygen species (ROS) namely singlet oxygen (^1^O_2_), superoxide (O_2_^•–^), and hydroxyl radicals (^•^OH), as well as other transient reactive intermediates.^23^Figure 1b illustrates typical reaction sequences following the formation of triplet-excited states of CDOM (^3^CDOM*).^24,25^ Short-lived ^3^CDOM* intermediates initiate a cascade of reactions that is responsible for O_2_ consumption. Note that these reactions also depend on the O_2_ spin state. ^3^CDOM* reacts with ground state (triplet) dioxygen, ^3^O_2_, to O_2_^•–^, and more reduced ROS as well as with organic carbon (shown in Figure 1b as C–H) to carbon-centered radicals (C^•^). Both processes ultimately consume ^3^O_2_ through the formation of water and oxygenated carbon species (e.g., carbonly and alcohol species). Alternatively, reactions of ^3^O_2_ with ^3^CDOM* results in transient ^1^O_2_. ^1^O_2_ species also contribute to further reductive oxygen elimination, on the one hand, through oxygenation reactions such as the formation of endo- and hydroperoxides with olefinic and aromatic compounds.^26^ On the other hand, ^1^O_2_ enables the one-electron oxidation of organic compounds and formation of O_2_^•–^^3,27,28^ and oxygenation of organic carbon through ROS generated from O_2_^•–^. Abiotic photochemical O_2_ consumption thus happens in a series of oxidation and oxygenation reactions that involve both ^3^O_2_ and ^1^O_2_ species. However, changes of O isotope ratios in O_2_ from O_2_ loss processes in aquatic environments have so far been determined exclusively for reactions of ^3^O_2_.^29−34^ It, therefore, remains unclear whether consumption of O_2_ through reactions of ^1^O_2_ exerts any effect on the observable ^18^O/^16^O and ^17^O/^16^O ratios of O_2_.

Changes of O isotope ratios of O_2_, also referred to as O isotope fractionation, occur because of kinetic isotope effects (KIEs) of (bio)chemical reactions, that is the different rate constants pertinent to the transformation of heavy (i.e., ^18^O/^16^O or ^17^O/^16^O) and light (^16^O^16^O) dioxygen isotopologues. This phenomenon is quantified in terms of O isotopic enrichment factors, O-ϵ. For changes of ^18^O/^16^O ratios, for example, the ^18^O-ϵ follows from eq 1.
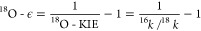
1where ^16^*k* and ^18^*k* are the rate constants for reactions of ^16^O^16^O and ^18^O^16^O isotopologues, respectively, and ^18^O-KIE is the corresponding kinetic isotope effect. Note that the same definition applies to ^17^O-ϵ and ^17^O-KIE for reactions of the ^16^O^16^O and ^17^O^16^O isotopologues. O-ϵ values reflect how bonds in O_2_ are broken and formed and are hence specific for the type of reaction or process responsible for O_2_ consumption.^35^^18^O-ϵ values for O_2_ respiration by aquatic microbial communities, in fact, are confined to a narrow range between −17‰ and −21‰.^29−31^ These numbers correspond to apparent ^18^O-KIEs of approximately 1.02. Conversely, ^18^O-ϵ values for abiotic photochemical O_2_ consumption determined with selected CDOM species of different origins generally amount to −6‰ to −12‰, with one distinct value at −22‰.^32−34^ The apparent difference of most of these ^18^O-ϵ values to data for respiration is key to disentangle competing oxygen consumption processes in the biogeochemical O_2_ cycling. Furthermore, the variability in reported ^18^O-ϵ values for abiotic photochemical O_2_ consumption might be caused by different relative contributions of alternative reaction pathways outlined in Figure 1b.

Contributions of reactions of ^1^O_2_ to the observed ^18^O-ϵ values for abiotic photochemical O_2_ consumption were not elucidated so far. Given that the photochemistry of ^1^O_2_ is well-understood,^3,23,24,36−40^ the isotope fractionation of O_2_ tied to the fate of ^1^O_2_ can be rationalized with two main processes (Figure 1b). First, the reversible formation of ^1^O_2_ from energy transfer of ^3^CDOM* to ^3^O_2_ and ^1^O_2_ decay back to ^3^O_2_. Second, chemical reactions of ^1^O_2_ through oxygenation of susceptible moieties of CDOM or other organic compounds and ^1^O_2_ reduction to O_2_^•–^. Isotope fractionation of reactants in such pre-equilibrium kinetic regimes are well understood from studies of enzyme kinetics.^41,42^ Such analyses of pre-equilibrium kinetics and isotope fractionation allow one to postulate that the maximum observable O_2_ isotope fractionation will be determined primarily by the isotope effects pertinent to ^1^O_2_ loss reactions, that is oxygenation to peroxide products and electron transfer to O_2_^•–^. However, the expression of isotope fractionation in O_2_ will critically depend on the ratio of rates of ^1^O_2_ decay to ^3^O_2_ vs oxygenation and electron transfer. As is known from reactions of enzymatically activated dioxygen species, such oxygenation and electron transfer reactions can exhibit substantial ^18^O-KIEs of up to 1.05.^43−50^

The goal of this work was to explore the magnitude and variability of isotope fractionation of reactions of O_2_ through the photochemical formation of ^1^O_2_. To that end, we studied O_2_ isotope fractionation associated with the formation of ^1^O_2_ and the reaction of ^1^O_2_ with reactive functional groups present in dissolved organic matter in laboratory model systems with probe compounds for ^1^O_2_-reactive CDOM moieties of known reactivity toward ^1^O_2_. Specifically, we (i) examined the kinetics of O_2_ consumption through ^1^O_2_-dependent pathways to identify kinetic regimes pertinent to hypothesized processes of reversible ^1^O_2_ formation vs irreversible ^1^O_2_ forward reactions. To that end, we established experimental conditions that enabled the analyses of O_2_ reaction kinetics and isotope fractionating processes in experiments with rose bengal, a well-studied ^1^O_2_ sensitizer, as well as three different amino acids (histidine, tyrosine, methionine) and furfuryl alcohol as chemical quenchers of ^1^O_2_. The quenchers, to which we refer to in the following as probe compounds, exhibit furan, imidazole, phenol, and sulfide moieties with documented reactivity with ^1^O_2_.^3,51^ (ii) We determined ^18^O-ϵ values for ^1^O_2_ formation from O_2_ and the ensuing reactions of ^1^O_2_ with various probe compounds and experimental conditions. (iii) Finally, we evaluated the ^18^O-KIEs of ^1^O_2_ reactions with probe compounds and discuss implications for applying O isotope analysis of O_2_ for assessment of photochemical O_2_ consumption.

## Materials and Methods

### Chemicals and Solutions

Unless noted otherwise, chemicals were purchased from Sigma-Aldrich and used as received. Furfuryl alcohol (98%), l-histidine (99%), l-methionine (99.5%), and l-tyrosine (99%) were used as probe compounds and rose bengal (95%) was used as a ^1^O_2_ sensitizer. Furfuryl alcohol was distilled prior to use. Sodium dihydrogen phosphate dihydrate (NaH_2_PO_4_·2H_2_O, 99%, Merck KGaA), dipotassium phosphate anhydrous (K_2_HPO_4_, 99%, Merck KGaA), hydrochloric acid (HCl, 37%, VWR Chemicals), and sodium hydroxide (NaOH, 98%) were used for making buffer solutions. Sodium sulfite anhydrous (Na_2_SO_3_, 98%) was used to calibrate optical oxygen sensors. All solutions were prepared in air-equilibrated ultrapure water (18.2 MΩ·cm, ELGA LabWater). He (99.999%), N_2_ (99.999%), and O_2_ (99.995%) were obtained from Carbagas.

### Irradiation Experiments

Irradiation experiments were performed in completely filled 12 mL Exetainer vials (15.5 mm o.d., Labco Limited) sealed with screw caps and butyl rubber septa or 12 mL crimp-top vials (22.5 mm o.d.) sealed with butyl rubber stoppers and aluminum crimp caps. All experiments were performed in 10 mM phosphate buffer at pH 7.0 (furfuryl alcohol), 7.7 (histidine), or 8.4 (methionine, tyrosine). The pH was chosen to match experimental conditions under which second-order reaction rate constants of the probe compounds with ^1^O_2_ were determined.^52−54^^1^O_2_ was selectively generated by visible light irradiation of rose bengal (0.4–20 μM). A 30 W fluorescent light bulb or an overhead projector were used as an irradiation source at a distance of 10–40 and 500–2000 cm, respectively. Initial probe compound concentrations were 0.25–250 mM for furfuryl alcohol, 0.15–100 mM for histidine, 0.2–1.2 mM for tyrosine, and 0.6–19 mM for methionine. For each set of conditions, 6–10 completely filled (i.e., headspace-free) and closed reactors containing buffer solution, probe compound, and rose bengal were irradiated for different amounts of time (up to 240 min) until desired final dissolved O_2_ concentrations of 25–265 μM were reached. Control experiments in which no O_2_ was consumed were performed without the addition of rose bengal or probe compounds. Dark controls were performed with reactors wrapped in aluminum foil. All controls were irradiated for the maximum duration of a given experiment. After irradiation, dissolved O_2_ concentrations were measured with an optical oxygen microsensor (PyroScience or PreSens–Precision Sensing), which was introduced into the closed reactors through a needle.^55^ O_2_ concentration measurements were temperature corrected, and the sensors were calibrated with air-saturated water and with a 300 mM Na_2_SO_3_ solution.

### Oxygen Isotope Analysis

^18^O/^16^O ratios of dissolved O_2_ were measured by gas chromatography coupled to isotope ratio mass spectrometry (GC/IRMS) as described previously.^49,56^ Briefly, a headspace was created with He (5 mL liquid replaced in Exetainer, experiments with fluorescence light bulb as light source) or N_2_ (3 mL liquid replaced in crimp-top vials, experiments with overhead projector as light source) in all reactors. Partitioning of O_2_ into the gas phase was facilitated by horizontal shaking at 200 rpm for 30 min (crimp-top vials) or 60 min (Exetainer), while the vials were kept upside down. Blank samples were prepared by filling vials completely with N_2_-purged water in an anaerobic glovebox with a N_2_ atmosphere (MBraun, residual O_2_ content 0.1 ppm or GS GLOVEBOX Systemtechnik, residual O_2_ content 1 ppm). Vials filled with air-equilibrated water and Exetainer containing 150 μL of ambient air in He were used as isotopic O_2_ reference standards. Blanks and standards containing water were prepared for analysis as described above for the samples from irradiation experiments. Most samples were analyzed by the GasBench/IRMS setup described in Bopp et al.^49^ with a 60 m Rt-Molsieve 5 Å PLOT column (Restek from BGB Analytik) at 25 °C. Some samples from experiments with furfuryl alcohol were analyzed by the GC/IRMS setup described in Pati et al.^56^ with a 30 m Rt-Molsieve 5 Å PLOT column at 30 °C. Despite potential for Ar interference in the latter setup,^49^ an identical experiment analyzed on both instruments gave identical results (see entries 3 and 4 in Table 1).

**Table 1:** Experimental Conditions, Probe Compound (PC Where FFA, His, Tyr, and Met Stand for Furfuryl Alcohol, Histidine, Tyrosine, and Methionine, Repectively) Type and Concentration, Rose Bengal (RB) Concentration, Light Source, Number of Replicates, Reaction Order, Rate Constants, Isotope Fractionation (^18^O-ϵ), and Apparent ^18^O-KIEs for all Experiments Conducted^a^

entry	PC	[PC] (mM)	[RB] (μM)	light source conditions^b^	replicates	kinetic order	d[O_2_]/d*t* (μM s^–1^) or (10^–3^ s^–1^)^c^	*k*_3_[PC]/*k*_2_ (−)	^18^O-ϵ (‰)	^18^O-KIE(−)
1	FFA	250	4	OHP	3	zero-order	0.40 ± 0.07	120	0.5 ± 0.7	0.9995 ± 0.0007
2	FFA	2.5	4	OHP	1	zero-order	0.24 ± 0.02	1.2	–16 ± 5	1.016 ± 0.005
3	FFA	0.25	4	OHP	1	first-order	0.46 ± 0.08	0.12	–21 ± 4	1.022 ± 0.004
4	FFA	0.26	10	FLB (10 cm)	1	first-order	0.56 ± 0.05	0.13	–23 ± 1	1.024 ± 0.002
5	His	100	0.4	FLB (20 cm)	1	zero-order	0.103 ± 0.006	26	–3.9 ± 0.5	1.0039 ± 0.0005
6	His	40	4	FLB (40 cm)	2	zero-order	0.180 ± 0.007	10	–7.0 ± 0.4	1.0070 ± 0.0004
7	His	15	0.5	FLB (20 cm)	1	zero-order	0.099 ± 0.008	3.8	–11.3 ± 0.8	1.0114 ± 0.0008
8	His	5.0	1	FLB (20 cm)	1	zero-order	0.138 ± 0.008	1.3	–18.5 ± 0.6	1.0189 ± 0.0006
9	His	4.0	4	FLB (20 cm)	4	zero-order	0.21 ± 0.01	1.0	–18.2 ± 0.7	1.0185 ± 0.0007
10	His	1.3	8	FLB (10 cm)	1	zero-order	0.34 ± 0.04	0.32	–20 ± 1	1.021 ± 0.001
11	His	0.40	4	FLB (10 cm)	5	zero-order	0.081 ± 0.003	0.10	–22.8 ± 0.5	1.0233 ± 0.0005
12	His	0.30	10	FLB (10 cm)	3	first-order	0.43 ± 0.03	0.077	–25.2 ± 0.4	1.0258 ± 0.0004
13	His	0.15	20	FLB (10 cm)	1	first-order	0.17 ± 0.02	0.038	–25 ± 2	1.026 ± 0.002
14	Tyr	1.2	20	FLB (10 cm)	3	zero-order	0.066 ± 0.005	0.038	–26.7 ± 0.9	1.0275 ± 0.0009
15	Tyr	0.43	20	FLB (10 cm)	2	zero-order	0.024 ± 0.001	0.014	–25 ± 1	1.025 ± 0.001
16	Tyr	0.20	20	FLB (10 cm)	2	first-order	0.061 ± 0.004	0.0064	–23.5 ± 0.5	1.0240 ± 0.0006
17	Met	19	1	FLB (10 cm)	2	zero-order	0.189 ± 0.006	1.2	–24.3 ± 0.8	1.0249 ± 0.0009
18	Met	1.9	10	FLB (10 cm)	1	zero-order	0.166 ± 0.008	0.12	–28.7 ± 0.7	1.0295 ± 0.0007
19	Met	1.2	10	FLB (10 cm)	2	zero-order	0.089 ± 0.003	0.77	–28.4 ± 0.9	1.029 ± 0.001
20	Met	0.6	20	FLB (10 cm)	3	zero-order	0.046 ± 0.003	0.038	–28 ± 1	1.029 ± 0.001
21	Met	0.3	20	FLB (10 cm)	1	zero-order	0.0213 ± 0.0009	0.019	–30 ± 1	1.031 ± 0.001

a Errors are given as 95% confidence intervals.

b OHP: overhead projector at variable distances, FLB: fluorescent light bulb at fixed distance.

c μM s^–1^ for zero-order kinetics and s^–1^ for pseudo-first order kinetics.

### Data Evaluation

^18^O/^16^O ratios are reported as δ^18^O values in per mil (‰, ± standard deviation) relative to Vienna Standard Mean Ocean Water (VSMOW). All δ^18^O values were corrected for blank contributions as described in Pati et al.^56^ as well as for instrument linearity (change in δ^18^O values with signal size) and instrument drift (change in δ^18^O values over time).^57^ Instrument linearity correction was based on daily measurements of reference gas peaks with different amplitudes, and instrument drift correction was based on measurements of standards evenly spread across each measurement sequence. Additionally, a one-point calibration with dilute air standards was applied assuming a δ^18^O value of 23.8‰ for O_2_ in ambient air.^58−61^ We recently showed that for the range of δ^18^O values measured in this study, such a one-point calibration provide sufficiently accurate ^18^O/^16^O ratios.^62^

The magnitude of O isotope fractionation associated with O_2_ consumption is reported in terms of ϵ value (in ‰, ±95% confidence intervals), which was calculated as the slope of a linear regression according to eq 2.
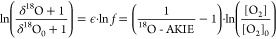
2where δ^18^O_0_ and δ^18^O are the O isotopic composition of O_2_ at the beginning of an experiment and in a sample at a given fraction of remaining O_2_ (), respectively, and ^18^O-AKIE is the apparent ^18^O kinetic isotope effect averaged for the two O atoms in O_2_.

## Results and Discussion

### Kinetics of Dissolved O_2_ Consumption through Reactions Involving ^1^O_2_

**Figure 2:**
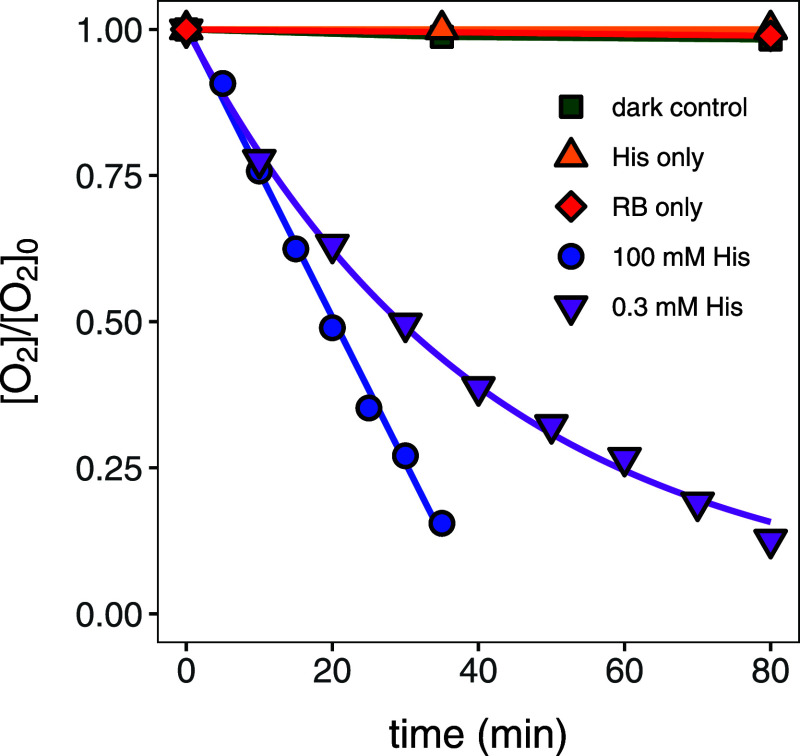
Normalized dissolved O_2_ concentrations vs time during irradiation experiments in aqueous solution with rose bengal (RB) and 0.3 mM histidine (His, purple downward triangles, entry 12 in Table 1), rose bengal and 100 mM histidine (blue circles, entry 5), histidine only (yellow upward triangles), rose bengal only (red diamonds), and during dark controls (green squares).

We examined the kinetics of O_2_ consumption through ^1^O_2_-dependent pathways to identify kinetic regimes pertinent to hypothesized processes of reversible ^1^O_2_ formation vs irreversible ^1^O_2_ reactions with ^1^O_2_ quenchers, that is organic probe compounds as postulated in Figure 1b. Typical concentration gradients of O_2_ from experiments, in which ^1^O_2_ is generated through irradiation of the sensitizer (rose bengal) and reacts with histidine as a probe compound, are shown in Figure 2. Experimental conditions (i.e., different probe compound and rose bengal concentrations, distance to light source, sampling intervals) were optimized so that 50–90% of the initial, dissolved O_2_ (approximately 270 μM) was consumed during total irradiation times of 16 to 240 min.

Figure 2 emblematically illustrates three typical observations for experiments with histidine as a probe compound. O_2_ reaction kinetics were zero-order at an initial histidine concentration that exceeded that of O_2_ substantially (100 and 0.3 mM for histidine and O_2_, respectively). Conversely, O_2_ consumption kinetics were pseudo-first order when similar initial concentrations of probe compound and O_2_ were used. Finally, only negligible aqueous O_2_ disappearance (i.e., < (2–6)% of the initial O_2_ concentration) was observed in control experiments without rose bengal, without probe compound, and in dark controls, where vials containing both rose bengal and probe compounds were wrapped in aluminum foil, respectively. The complete compilation of experimental conditions, observed O_2_ reaction order, and O_2_ disappearance rate constants for experiments with different probe compounds are shown in Table 1. Note that O_2_ consumption in experiments with methionine followed apparent zero-order kinetics given that initial precursor compound concentrations exceeded the dissolved O_2_ saturation concentration in all experimental conditions. This observation is consistent with results from experiments with elevated concentrations of other probe compounds.

We rationalized the observed reaction order of O_2_ disappearance using a rate law expression in which we assume that O_2_ reversibly forms ^1^O_2_. The latter subsequently undergoes irreversible reaction(s) with one of the probe compounds as in eq 3.
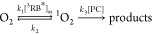
3where *k*_1_, *k*_2_, and *k*_3_ are reaction rate constants for ^1^O_2_ formation from excited triplet states of rose bengal (^3^RB*), the deactivation of ^1^O_2_ to ^3^O_2_, and any oxygenation or electron transfer reaction of ^1^O_2_ with the probe compound, respectively. [^3^RB*]_ss_ is the steady-state concentration of triplet-excited states of rose bengal in the irradiation experiments, and [PC] stands for the concentration of the probe compound. Note that numerical values for *k*_2_ and *k*_3_ have been determined previously.^52−54,63^

Following the kinetic scheme of eq 3, the disappearance of O_2_ in our experiments is given by eq 4
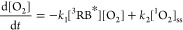
4where [O_2_] is the dissolved oxygen concentration and [^1^O_2_]_ss_ is the steady-state concentration of singlet oxygen, which follows from eq 5.
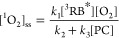
5

Substitution of eq 5 into eq 4 and some rearrangements lead to eq 6. This equation allows for expressing the rate of O_2_ removal in terms of the ratio of forward and backward reaction rates of ^1^O_2_ (*k*_3_[PC]/*k*_2_), respectively, and thus includes the concentration of the probe compound. The latter was a key factor determining the apparent reaction order of O_2_ reactivity.
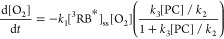
6

In our experimental setup, we can assume that the dominant quenching mechanism of ^3^RB* is the reaction with dissolved O_2_.^24^ Following the formalism used by Rosario–Ortiz and Canonica,^25^ [^3^RB*]_ss_ is then given by eq 7.
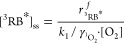
7where  is the rate of ^3^RB* formation and  is the singlet oxygen formation yield for the reaction between ^3^RB* and dissolved O_2_. Insertion of eq 7 into the rate law of O_2_ consumption, eq 6, leads to eq 8 that we use here to interpret the observed reaction order.
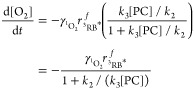
8

**Figure 3:**
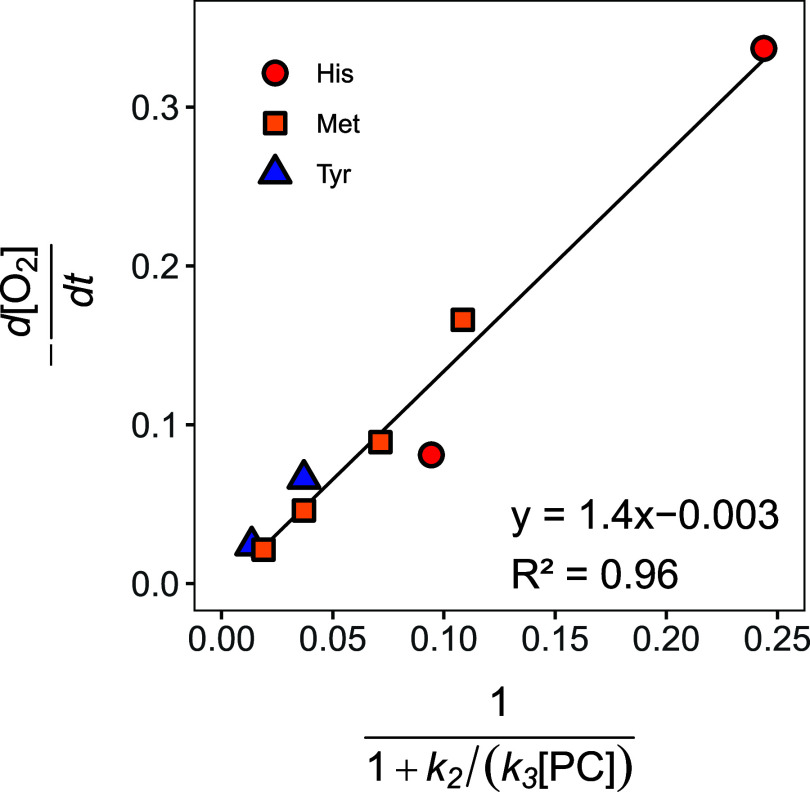
Linear correlation between apparent zero-order rate constants and the kinetic term 1/(1 + *k*_2_/(*k*_3_[PC])) in experiments with histidine (His, red circles), tyrosine (Tyr, blue triangles), and methionine (Met, yellow squares) performed under similar experimental conditions (see entries 10–11, 14–15, and 18–21 in Table 1).

Note that according to eq 8, the rate of O_2_ consumption is now independent of [O_2_]. Kinetics of O_2_ consumption follow zero order for large [PC] concentrations when the denominator approaches unity, that is 1 + *k*_2_/(*k*_3_[PC]) ≈ 1. The rate of O_2_ consumption is then determined by ^1^O_2_ formation and equals . In fact, in all experiments with identical light conditions (fluorescent light bulb at 10 cm distance; FLB (10 cm) in Table 1) and rose bengal concentrations between 4 and 20 μM, apparent zero-order rate constants linearly correlated with 1/(1 + *k*_2_/(*k*_3_[PC])) as shown in Figure 3. According to eq 8, the slope of the linear regression in Figure 3 is the rate of ^1^O_2_ formation, , which appears constant under these experimental conditions. Apparent zero-order rate constants obtained with different light source conditions or with lower rose bengal concentrations do not show this linear correlation. This observation indicates that different rates of ^1^O_2_ formation for these cases were likely due to a different . This interpretation of the observed O_2_ disappearance supports the validity of the rate law from eq 8, when [PC] is sufficiently large to be considered constant throughout the experiment. Once [PC] becomes small, eq 8 indicates the change from zero- to first-order kinetics, as we observed with probe compounds furfuryl alcohol, histidine, and tyrosine. Under these conditions, the rate of O_2_ consumption is limited by reactions of ^1^O_2_ with the probe compound and linearly depends on [PC]. Finally, if the initial probe compound concentrations and that of dissolved O_2_ are of similar magnitude and decrease at similar rates during the course of the reaction, then both ^1^O_2_ formation equilibria and irreversible forward reaction with the probe compound contribute to the observed rate of O_2_ disappearance. The reaction kinetics will then appear to be first order (see entries 3, 4, 12, 13, and 16 in Table 1). Collectively, the kinetic analysis with eq 8 allows for disentangling the reversible ^1^O_2_ formation process and the irreversible reaction of ^1^O_2_ with the probe compounds. The outcome is also critical to assign any observed O isotope fractionation of O_2_ to the two processes.

### Isotope Fractionation of Dissolved O_2_ through Reactions with ^1^O_2_

**Figure 4:**
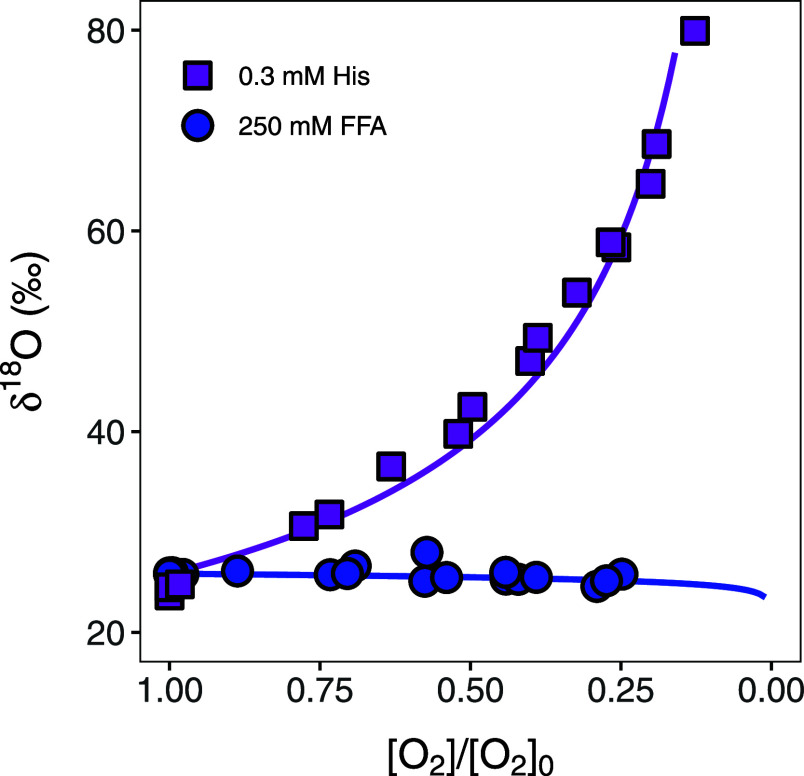
Change in δ^18^O values vs fraction of remaining O_2_ concentration ([O_2_]/[O_2_]_0_) during irradiation experiments with rose bengal and 0.3 mM histidine (His, purple squares, entry 12 in Table 1) and 250 mM furfuryl alcohol (FFA, blue circles, entry 1).

Typical trends of δ^18^O values of dissolved O_2_ during irradiation experiments with rose bengal and probe compounds are shown in Figure 4. Substantial O isotope fractionation is illustrated with histidine as a probe compound at an initial concentration of 0.3 mM (entry 12, Table 1). δ^18^O values increased from 23.6 ± 0.2‰ to 79.9 ± 0.8‰ as 87% of the initial, dissolved O_2_ was consumed, corresponding to an ^18^O-ϵ of −25.5‰. Conversely, experiments with furfuryl alcohol as probe compound at an initial concentration of 250 mM revealed only minor O isotope fractionation, that is a change in δ^18^O values from 25.8‰ to 28.0‰ despite a similar degree of O_2_ consumption (entry 3, Table 1). Note that the maximum change of δ^18^O observed in a control experiment was 0.7‰ which corresponds to the equilibrium isotope fractionation for gaseous O_2_ dissolution in water^1^ and was used here as lower bound for negligible isotope fractionation comparison. To that end, all of the photochemical experiments shown here reveal some extent of O isotope fractionation which we can thus ascribe to the reaction of dissolved O_2_ with triplet excited states of rose bengal, the formation of singlet oxygen, and the subsequent reaction between ^1^O_2_ and probe compounds.

The data in Table 1 show that the extent of O isotope fractionation and thus ^18^O-ϵ vary substantially from no fractionation (0.5 ± 0.7)‰ to −30‰ for all probe compounds studied. As a general trend, O isotope fractionation increases (i.e., ^18^O-ϵ values become more negative) with decreasing initial probe compound concentration. The extent of O isotope fractionation, however, did not correlate with reaction order. An interpretation of the observed trends of ^18^O-ϵ values follows from the evaluation of isotope effects pertinent to the elementary reaction steps of O_2_ activation to ^1^O_2_ and its reaction with the probe compounds outlined in eq 3.

### Derivation of Isotope Effects Associated with the Formation and Reactions of ^1^O_2_

We derived the isotope effects of ^1^O_2_ formation and subsequent oxygenation or electron transfer reactions from the isotopic expression of eq 6 as documented in Section S1 of the Supporting Information. To that end, we consider the ratio of O_2_ isotopologue disappearance for the two most abundant isotopologues, ^16^O^16^O and ^18^O^16^O, to obtain the apparent ^18^O-kinetic isotope effect, ^18^O-AKIE, in eq 10 from the ratio of the apparent rate constants ^16^*k*_obs_ and ^18^*k*_obs_.
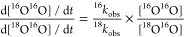
9
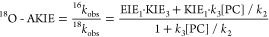
10where [^16^O^16^O] and [^18^O^16^O] are the considered isotopologue concentrations, respectively, subscripts 1 to 3 denote the elementary processes from eq 3, EIE_1_ is the equilibrium isotope effect of the reversible ^1^O_2_ formation. KIE_1_ and KIE_3_ are the kinetic isotope effect of ^1^O_2_ formation and the irreversible reaction of ^1^O_2_ with the probe compound. *k*_3_[PC]/*k*_2_ describes the ratio of the forward and backward reaction of ^1^O_2_ and, therefore, the relevance of reactions with probe compounds for elimination of O_2_. The right-hand term in eq 10 is typical for describing the modulation of isotope effects in enzymatic pre-equilibrium kinetics where *k*_3_[PC]/*k*_2_ would be equivalent to a (forward) commitment factor in a catalytic reaction.^41,42^

We converted the observable O isotope fractionation which one quantifies with ^18^O-ϵ into ^18^O-AKIE values by making use of the different reactivity of the probe compounds with ^1^O_2_. Under conditions that favor ^1^O_2_ elimination through reactions with the probe compounds, that is at high *k*_3_[PC]/*k*_2_, eq 10 simplifies to KIE_1_. As shown in experiments with high furfuryl alcohol concentration where *k*_3_[PC]/*k*_2_ = 120 (entry 1 in Table 1), the ^18^O-AKIE then corresponds to unity and indicates the absence of O isotope fractionation. This observation implies that the KIE_1_ also corresponds to 1.0. This interpretation is supported by the fact that O isotope fractionation of O_2_ reactions with minor oxygen bonding changes, as in the formation of ^1^O_2_, are indeed small and EIE_1_, KIE_1_, and KIE_2_ values equal to unity.^44^ Finally, KIE_1_, KIE_2_, and, therefore, EIE_1_ are likely identical and independent of the reaction with any of the probe compounds in our experiments because only O_2_, rose bengal, and water are involved in the first reaction step leading to ^1^O_2_ formation and decay (see eq 3). Following this logic, we are able to deduce the magnitude of KIE_3_ from experiments where *k*_3_[PC]/*k*_2_ ≪ 1 and ^18^O-AKIE then corresponds to KIE_3_.

### Magnitude of Apparent Kinetic Isotope Effects of O_2_ Consumption through the Photochemical ^1^O_2_ Formation Pathway

**Figure 5:**
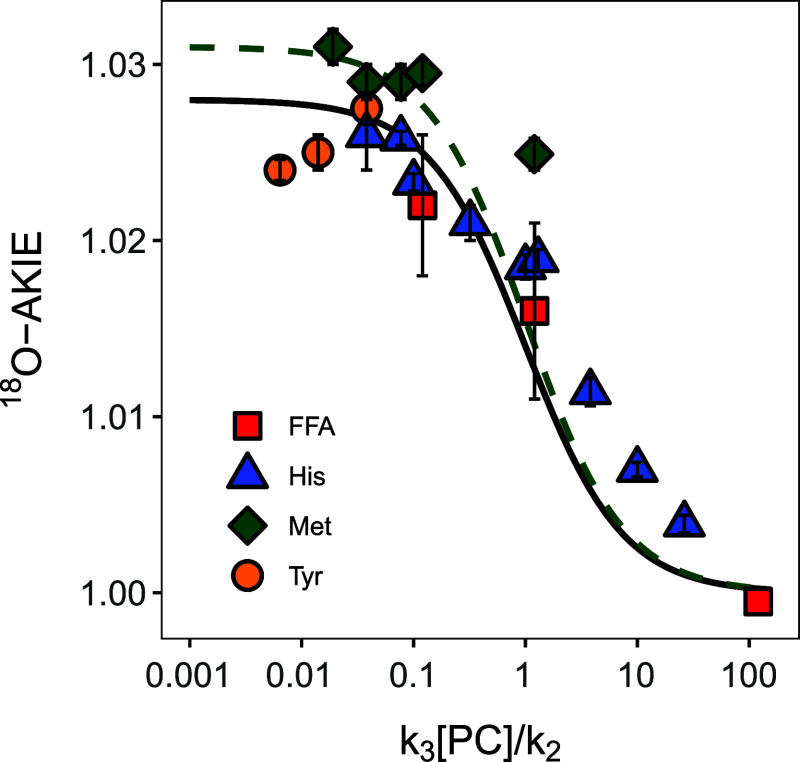
Apparent ^18^O-KIEs vs *k*_3_[PC]/*k*_2_ (see Table 1) from experiments with furfuryl alcohol (FFA, red squares), histidine (His, blue triangles), methionine (Met, green diamonds), and tyrosine (Tyr, yellow circles). The black solid and green dashed lines were calculated with eq 10, a KIE_1_ of 1, and KIE_3_ values of 1.028 and 1.031, respectively. Note that the *x*-axis has a logarithmic scale for improved resolution of the data points.

The ^18^O-AKIE values derived from ^18^O-ϵ with eq 2 of the experiments shown in Table 1 are plotted in Figure 5 vs the ratio of forward and backward reactions of ^1^O_2_, *k*_3_[PC]/*k*_2_. Regardless of the experimental conditions and probe compounds used for quenching ^1^O_2_, ^18^O-AKIE values follow the general trend outlined in eq 10 with the assumption that the reversible formation of ^1^O_2_ from the reaction of O_2_ with ^3^RB* does not exhibit any O isotope fractionation. Therefore, isotope effects associated with O_2_ disappearance reflect the reaction of ^1^O_2_ with the probe compounds. The O isotope fractionation of O_2_ of these reactions are masked (i.e., ^18^O-AKIE ≈ 1) at high *k*_3_[PC]/*k*_2_, that is in kinetic regimes of so-called forward commitment, where the forward reaction of ^1^O_2_ out-competes its decay to O_2_. Conversely, ^18^O-AKIE values approach a maximum value of 1.028 and 1.031 (solid and dashed lines in Figure 5) and thus the intrinsic kinetic isotope effect (KIE_3_) when the ^1^O_2_ formation/decay equilibrium is faster than ^1^O_2_ oxygenation and electron transfer.

We note, however, small deviations of this general trend for the different probe compounds used. In experiments with methionine, ^18^O-AKIE values approach a higher intrinsic KIE_3_ of 1.031 (dashed line in Figure 5) than that with histidine (1.026, solid line). ^18^O-AKIEs from experiments with tyrosine even decrease slightly from 1.0275 to 1.0240 as forward commitment decreases (Table 1). Data for ^18^O-AKIEs for furfuryl alcohol, unfortunately, only extend to *k*_3_[PC]/*k*_2_ of 0.12 and thus do not allow for observation of the maximum O isotope fractionation. Moreover, some ^18^O-AKIEs from reactions with histidine and methionine are approximately 0.002 to 0.003 AKIE units higher than rationalized with eq 10 for *k*_3_[PC]/*k*_2_ values between 0.3 and 10. We hypothesize that the observed deviations from trends rationalized by eq 10 have two possible origins; distinct reaction mechanisms of ^1^O_2_ attack at the probe compounds and, possibly, different concentrations of ^1^O_2_ across the various experiments.

In fact, based on isotopic pre-equilibrium models underlying eq 10, we assume constant concentrations of ^1^O_2_ and ^3^RB* over the course of an experiment, reactions of O_2_ and ^3^RB* as the predominant ^3^RB* quenching mechanism, as well as a constant rate of formation of ^3^RB* and a constant ^1^O_2_ formation yield. Given that the rose bengal concentrations varied between 0.5 and 20 μM, the assumptions implied in the pre-equilibrium model are likely not strictly valid for all experimental conditions. The ensuing differences in reaction kinetics could be potential sources of the observed variations of the ^18^O-AKIE values.

Histidine, tyrosine, and furfuryl alcohol share a common initial reaction step through cycloaddition of ^1^O_2_ to the imidazole, phenyl, and furan moieties of the probe compounds.^40,54^ It is thus reasonable to assume that the similar ^18^O-AKIE values for O_2_ consumption and consistent trends with *k*_3_[PC]/*k*_2_ originate from an intrinsic KIE_3_ of similar magnitude within the uncertainty of 0.003 AKIE-units identified here (Figure 5). ^18^O-AKIEs from experiments with methionine, by contrast, are offset from the other three probe compounds. One possible explanation for this offset is that the initial reaction step of methionine with ^1^O_2_ is indeed different. Methionine reacts with ^1^O_2_ to a persulfoxide species,^40^ where only one O atom of ^1^O_2_ participates in S–O bond formation instead of both O atoms in cycloaddition mechanisms. Such correlations of decreasing changes in O bond order with decreasing experimental and theoretical O isotope effects have been established for thermal reduction reactions of O_2_.^43^ Moreover, the maximum AKIE determined in experiments with methionine (1.031, entry 21 in Table 1) is in good agreement with the calculated equilibrium isotope effect for O_2_ reduction to HO_2_^–^ of 1.034.^43^ Unfortunately, isotope effect calculations for ^1^O_2_ additions to olefins, which might provide estimates for ^18^O-AKIE values for experiments with tyrosine, have been restricted to C and H isotopes of the olefinic probe compounds.^64^ A comparison of our data with theoretical ^18^O isotope effects for ^1^O_2_ reactions is thus not possible. Regardless of the limited availability of theoretical isotope effect data for comparison, the reaction mechanisms leading to the oxygenation of the studied probe compounds with ^1^O_2_ can be more complicated than the simple kinetic scheme assumed here (eq 3), for example, by including competing reactions and multiple rate-limiting steps and, thus, representing a likely source of AKIE variability.

## Conclusions

Our data show that photochemical O_2_ consumption through pathways involving ^1^O_2_ can lead to significant O isotope fractionation that originates from oxygenation reactions of organic compounds with ^1^O_2_. In surface waters, such compounds are present as reactive structural moieties of DOM. The magnitude of isotope fractionation of dissolved O_2_ will thus dependend on the concentration, type, and accessibility of reactive DOM moieties. At high concentrations of DOM or ^1^O_2_-reactive moieties therein, we expect a similar masking of O kinetic isotope effects through fast reactions of ^1^O_2_ that would result in only negligible, observable O isotope fractionation of dissolved O_2_. With decreasing concentrations of reactive functional groups, ^18^O-ϵ values of dissolved O_2_ will increase to up to −27‰ to −30‰ once the concentration of furan, imidazole, phenol, and sulfide moieties becomes kinetically limiting. This increase in O isotope fractionation of dissolved O_2_ is caused by a shift in the rate-limiting step of the overall reaction from the formation of ^1^O_2_ (no isotope fractionation) to the oxygenation and electron transfer reaction between ^1^O_2_ and organic moieties.

While the exact nature of ^1^O_2_-reactive functional groups in natural organic matter is still unclear, phenolic moieties, for example, in lignin-derived molecules,^65^ are likely the most abundant ^1^O_2_-reactive functional group with concentrations in terrestrial dissolved organic matter of 2–4 mmol g^–1^ C.^66^ With typical dissolved organic carbon concentrations of 0.5–3 mg L^–1^ for oceans and 0.5–50 mg L^–1^ for lakes and rivers,^67^ the total concentration of phenolic moieties ranges between 1 and 200 μM. Our work shows that the magnitude of O isotope fractionation increases with decreasing probe compound concentration. Given that we already observe a maximum ^18^O-AKIE at 200 μM, the lowest phenolic moiety concentration used in this study (as tyrosine), O isotope fractionation associated with photochemical O_2_ consumption due to ^1^O_2_ formation can be expected to be close to the −24‰ determined with 200 μM tyrosine. Consequently, isotope fractionation of photochemical O_2_ consumption through ^1^O_2_ formation differs from isotope fractionation of respiration. This comparison implies that photochemical O_2_ consumption should be integrated as part of the assessment of gross photosynthetic O_2_ production and net community O_2_ production through the evaluation of (triple) oxygen isotope fractionation of O_2_. The same conclusion was reached by Sutherland et al.^34^ who estimated that neglecting photochemical O_2_ consumption can lead to an underestimation of gross productivity of 15% assuming only 10% of O_2_ is consumed photochemically. Before doing so, however, the isotope fractionation of additional photochemical O_2_ consumption pathways through mechanisms of single electron transfer and oxygenation reactions needs to be assessed. This necessity becomes particularly obvious when comparing the range of isotope fractionation determined in this study for ^1^O_2_ formation specifically (0‰ to −30‰) and previously for overall photochemical O_2_ consumption by DOM (−6‰ to −12‰ in most cases).^32−34^ This comparison suggests that ^1^O_2_ formation might not be the dominant pathway for photochemical O_2_ consumption in most complex CDOM mixtures, a conclusion supported by previously observed photochemical O_2_ consumption of fulvic acid solutions.^65^

## Data Availability

The data underlying this study are openly available at 10.5281/zenodo.14267139.

## Supporting Information Available

Derivation of algebraic expression for ^18^O-AKIE for O_2_ consumption (PDF)
