# Supplementary material for: Oxygen Isotope Fractionation of O2 Consumption through Abiotic Photochemical Singlet Oxygen Formation Pathways
Source: ACS Environ Au. 2025 Jan 22;5(2):220–9. doi: 10.1021/acsenvironau.4c00107 (PMC11926750; doi:10.1021/acsenvironau.4c00107)
Supplement: Supplementary file 1 — vg4c00107_si_001.pdf [file vg4c00107_si_001.pdf]

# *Supporting Information*
# Oxygen Isotope Fractionation of $O_2$ Consumption Through Abiotic Photochemical Singlet Oxygen Formation Pathways

Sarah G. Pati,[*,1,2] Lara M. Brunner,[2] Martin Ley,[2] and Thomas B. Hofstetter[*,3,4]

[1]Department of Environmental Geosciences, Centre for Microbiology and Environmental Systems Science, University of Vienna, 1090 Vienna, Austria, [2]Department of Environmental Sciences, University of Basel, 4056 Basel, Switzerland, [3]Eawag, Swiss Federal Institute of Aquatic Science and Technology, 8600 Dübendorf, Switzerland, [4]Institute of Biogeochemistry and Pollutant Dynamics (IBP), ETH Zürich, 8092 Zürich, Switzerland

[*]Corresponding authors: `sarah.pati@univie.ac.at`, `thomas.hofstetter@eawag.ch`

3 Pages

## Contents

**S1 Derivation of expression for the apparent $^{18}$O kinetic isotope effects of $O_2$ consumption 2**

# S1 Derivation of expression for the apparent $^{18}$O kinetic isotope effects of $O_2$ consumption

The following step-by-step derivation leads to eq. 10 in the main manuscript. We start with the rate law of eq. S2 for pre-equilibrium kinetics depicted in eq. S1. The two expressions correspond to eqs. 3 and 6 in the main manuscript and are reproduced here for clarity.

$$O_2 \underset{k_2}{\overset{k_1[^3\mathrm{RB}^*]_{\mathrm{ss}}}{\rightleftarrows}} {}^1O_2 \xrightarrow{k_3[\mathrm{PC}]} \text{products} \tag{S1}$$

$$\frac{d[O_2]}{dt} = -k_1[^3\mathrm{RB}^*]_{\mathrm{ss}}[O_2]\left(\frac{k_3[\mathrm{PC}]/k_2}{1 + k_3[\mathrm{PC}]/k_2}\right) = -k_{\mathrm{obs}}[O_2] \tag{S2}$$

We consider the ratio of $O_2$ isotopologue disappearance for the two most abundant isotopologues, $^{16}O^{16}O$ and $^{18}O^{16}O$, to obtain the apparent $^{18}$O-kinetic isotope effect, $^{18}$O-AKIE in eq. S4 from the ratio of apparent rate constants ($^{16}k_{\mathrm{obs}}$ and $^{18}k_{\mathrm{obs}}$ in eq. S3).

$$\frac{d[^{16}O^{16}O]/dt}{d[^{18}O^{16}O]/dt} = \frac{^{16}k_{\mathrm{obs}}}{^{18}k_{\mathrm{obs}}} \cdot \frac{[^{16}O^{16}O]}{[^{18}O^{16}O]} \tag{S3}$$

$$^{18}\text{O-AKIE} = \frac{^{16}k_{\mathrm{obs}}}{^{18}k_{\mathrm{obs}}} = \frac{^{16}k_1[^3\mathrm{RB}^*] \cdot ^{16}k_3[\mathrm{PC}]/^{16}k_2}{(1 + ^{16}k_3[\mathrm{PC}]/^{16}k_2)} \cdot \frac{(1 + ^{18}k_3[\mathrm{PC}]/^{18}k_2)}{^{18}k_1[^3\mathrm{RB}^*] \cdot ^{18}k_3[\mathrm{PC}]/^{18}k_2} \tag{S4}$$

$^{16}k_j$ and $^{18}k_j$ denote reaction rate constant is for the light ($^{16}O^{16}O$) or heavy isotopologues ($^{18}O^{16}O$), respectively, whereas subscripts $j$ refer to the elementary reactions in eq. S1, namely formation of $^1O_2$ the reaction of $O_2$ with $^3$RB$^*$, $^1O_2$ decay to $O_2$, and $^1O_2$ reactions with probe compounds.

In a first step, we remove the concentration term [$^3$RB$^*$] and rearrange the rate constants to get eq. S5. Note that $^{16}k_j$ divided by $^{18}k_j$ equals $^{18}$O KIE$_j$, the intrinsic kinetic isotope effect associated with the elementary reaction step of $k_j$ in eq. S1. The superscript "18" of the KIE is ignored in the following equations for simplicity.

$$^{18}\text{O-AKIE} = \frac{\mathrm{KIE}_1 \cdot ^{16}k_3[\mathrm{PC}]/^{16}k_2 \cdot (1 + ^{18}k_3[\mathrm{PC}]/^{18}k_2)}{^{18}k_3[\mathrm{PC}]/^{18}k_2 \cdot (1 + ^{16}k_3[\mathrm{PC}]/^{16}k_2)} \tag{S5}$$

To get from eq. S5 to eq. S6, we divide the nominator and denominator each by $^{18}k_3[\mathrm{PC}]/^{18}k_2$. KIE$_1$/KIE$_2$ equals EIE$_1$, the equilibrium isotope effect of the reversible formation of $^1O_2$ from $O_2$.

$$^{18}\text{O-AKIE} = \frac{\text{EIE}_1 \cdot \text{KIE}_3 + \text{KIE}_1 \cdot {}^{16}k_3[\text{PC}]/{}^{16}k_2}{1 + {}^{16}k_3[\text{PC}]/{}^{16}k_2} \tag{S6}$$

$$\approx \frac{\text{EIE}_1 \cdot \text{KIE}_3 + \text{KIE}_1 \cdot k_3[\text{PC}]/k_2}{1 + k_3[\text{PC}]/k_2} \tag{10}$$

We remove superscripts "16" from expression for $k_2$ and $k_3$, assuming that the rate constants associated with the disappearance of the abundant, light isotopologue, $^{16}O^{16}O$, are approximately equal to the rates associated with the overall disappearance of $O_2$, that is of all O isotopologues. Equation S6 then becomes eq. 10 in the main manuscript.
